# Surefire infusion system versus standard microcatheter use during holmium-166 radioembolization: study protocol for a randomized controlled trial

**DOI:** 10.1186/s13063-016-1643-3

**Published:** 2016-10-25

**Authors:** Andor F. van den Hoven, Jip F. Prince, Rutger C. G. Bruijnen, Helena M. Verkooijen, Gerard C. Krijger, Marnix G. E. H. Lam, Maurice A. A. J. van den Bosch

**Affiliations:** Department of Radiology and Nuclear Medicine, University Medical Center Utrecht, Heidelberglaan 100, 3584 CX Utrecht, The Netherlands

## Abstract

**Background:**

An anti-reflux catheter (ARC) may increase the tumor absorbed dose during radioembolization (RE) by elimination of particle reflux and its effects on hemodynamics. Since the catheter is fixed in a centro-luminal position, it may also increase the predictive accuracy of a scout dose administration before treatment. The purpose of the SIM trial is to compare the effects of ARC use during RE with holmium-166 (^166^Ho) microspheres in patients with colorectal liver metastases (CRLM), with the use of a standard end-hole microcatheter.

**Methods/Design:**

A within-patient randomized controlled trial (RCT) will be conducted in 25 patients with unresectable chemorefractory liver-dominant CRLM. Study participants will undergo a ^166^Ho scout dose procedure in the morning and a therapeutic procedure in the afternoon. The ARC will be randomly allocated to the left/right hepatic artery, and a standard microcatheter will be used in the contralateral artery. SPECT/CT imaging will be performed for quantitative analyses of the microsphere distribution directly after the scout and treatment procedure. Baseline and follow-up investigations include ^18^F-FDG-PET + liver CT, clinical and laboratory examinations. The primary endpoint is the comparison of tumor to non-tumor (T/N) activity ratio in both groups. Secondary endpoints include comparisons of mean absorbed dose in tumors and healthy liver tissue, infusion efficiency, the predictive value of ^166^Ho scout dose for tumor response. In the entire cohort, a dose-response relationship, clinical toxicity, and overall survival will be assessed. The sample was determined for the expectation that the ARC will increase the T/N ratio by 25 % (mean T/N ratio 2.0 vs. 1.6).

**Discussion:**

The SIM trial is a within-patient RCT that will assess whether ^166^Ho RE treatment can be optimized by using an ARC.

**Trial registration:**

The SIM trial is registered at clinicaltrials.gov (NCT02208804). Registered on 31 July 2014.

**Electronic supplementary material:**

The online version of this article (doi:10.1186/s13063-016-1643-3) contains supplementary material, which is available to authorized users.

## Background

Hepatic radioembolization (RE) has evolved to a standard-of-care therapy for patients with irresectable and chemorefractory colorectal cancer liver metastases (CRLM). During this treatment, radioactive microspheres are injected in the hepatic arteries to irradiate liver tumors from within, leading to local disease control [1]. However, treatment efficacy and predictability of the treatment effect can still be optimized, since the radiation dose to individual tumors is often inadequate and this cannot be predicted beforehand due to the lack of a reliable surrogate particle (scout dose) for routinely used yttrium-90 (^90^Y) microspheres [2].

Holmium-166 (^166^Ho) microspheres have been developed and clinically validated as a new microsphere for RE [3]. Besides β-radiation, these microspheres also emit low-energy γ-radiation and have paramagnetic characteristics, allowing for visualization and quantitative assessment of the microsphere distribution on single-photon emission computed tomography (SPECT) and magnetic resonance imaging (MRI) [4]. Furthermore, identical ^166^Ho microspheres can be administered as a scout dose to predict the distribution of the therapeutic microspheres [3, 5].

Another promising development is the availability of an anti-reflux catheter (ARC, Surefire Infusion System, Surefire Medical Inc., Westminster, CO, USA), aiming to increase the infusion efficiency and safety, by prevention of reflux [6–8]. The effects on hemodynamics induced by this catheter may improve the microsphere uptake ratio between tumorous and non-tumorous liver tissue (T/N ratio). The expanded catheter tip decreases blood pressure downstream of the tip, which is believed to lower the resistance that injected microspheres need to overcome in order to reach the tumor vasculature [9]. Furthermore, the catheter tip converts blood flow from a laminar into a turbulent pattern, which reduces the chance of missing a target branch by proper mixing of the microspheres in the vascular compartment. In addition, the catheter is fixed into a centro-luminal catheter position during infusion which may further increase the accuracy of the ^166^Ho scout dose as a predictor for the therapeutic ^166^Ho microspheres distribution [2].

In this article, we describe the study protocol of a prospective, comparative, clinical trial with the aim to investigate whether the ARC can be used to improve tumor targeting as well as the predictability of the treatment effect during ^166^Ho RE.

## Methods

### Hypothesis

We hypothesize that the use of the ARC increases the posttreatment T/N activity ratio and improves the predictive value of ^166^Ho scout dose distribution, in comparison with the use of a standard microcatheter (SMC).

### Study design

The SIM trial is a single-center, open-label, phase II, within-patient randomized controlled trial (RCT). As opposed to a conventional RCT design, patients act as their own controls; liver lobes (right versus left lobe) are the experimental units in this study. Patients with bilateral, irresectable, chemorefractory, liver-dominant CRLM will undergo two sequential procedures on the same day, during which a scout dose and a therapy dose of ^166^Ho microspheres will be administered in the left and right hepatic artery. These separate injections can be regarded as two separate treatments of functionally independent liver lobes. The use of the ARC will be randomly allocated (1:1) to the left or right functional liver lobe and a standard microcatheter (SMC) will be used in the contralateral side. The same catheter type will be used on the same side during the scout and therapy procedures.

This trial will be conducted in accordance with the principles outlined in the Declaration of Helsinki and will follow the Consolidated Standards of Reporting Trials (CONSORT) statement [10]. A Standard Protocol Items: Recommendations for Interventional Trials (SPIRIT) checklist is provided in Additional file 1 [11].

### Study population

All patients with irresectable, chemorefractory, and liver-dominant CRLM are eligible for participation in this trial if they meet the following inclusion criteria: histopathologically confirmed diagnosis of adenocarcinoma of the colon or rectum, hepatic metastases with measurable morphological appearance (≥1 cm) on cross-sectional imaging located in both the right and left hepatic arterial perfusion territory, irresectable and liver-dominant disease (i.e., pathological locoregional lymph nodes and up to five lung lesions < 1 cm are accepted), progressive disease after standard second-line systemic treatment or no further systemic treatment options due to severe side effects or unwillingness of the patient, age ≥ 18 years. Adequate follow-up must be logistically feasible and written informed consent must be obtained before enrollment in the study.

Exclusion criteria are: World Health Organization (WHO) performance score > 2, inadequate bone marrow function (hemoglobin < 6.0 mmol/l, leukocyte count < 3.0 × 10^9^/l, platelet count < 75 × 10^9^/l), inadequate liver function (bilirubin > 35 μmol/l, aspartate aminotransferase/alanine aminotransferase > 5 × upper limit of normal) or inadequate renal function (creatinine > 1.5 × upper limit of normal), prior hemihepatectomy, compromised biliary system (biliary stent or hepaticojejunostomy), Child-Pugh score B7 or worse, active hepatitis B or C, main portal vein thrombosis or previous portal vein embolization, severe celiac axis stenosis, unsuitable hepatic arterial anatomy, treatment with systemic chemotherapy within 4 weeks prior to RE, previous participation in a study classified as class III by a radiation safety committee, bleeding diathesis, pregnancy or breast feeding, or any condition that prevents safe treatment with RE.

### Investigations and interventions

**Fig. 1:**
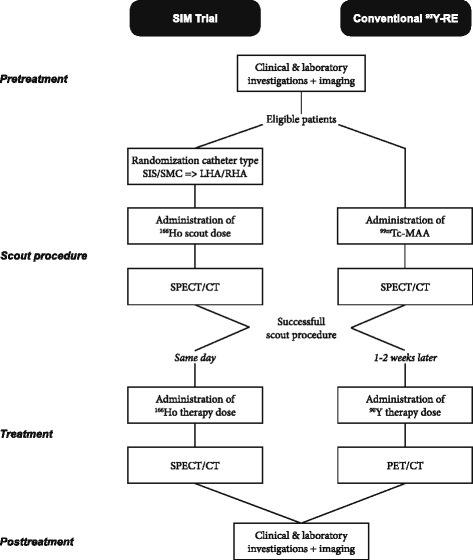
Flowchart of the investigations and interventions in the SIM trial. The study procedures are compared to standard radioembolization practice with yttrium-90 (^90^Y) microspheres. Note that in the SIM trial, the same particle is used during the scout and therapy procedure, and patients receive all procedures on the same day

The flowchart in Fig. 1 shows how the investigations and interventions in the SIM trial compare to standard radioembolization practice with ^90^Y microspheres.

#### Baseline investigations

Patients will first undergo pretreatment investigations as part of our routine RE workup: whole-body ^18^F-fluorodeoxyglucose positron emission tomography/computed tomography (^18^F-FDG-PET/CT) to rule out extensive extrahepatic metastases and calculate metabolic tumor burden; dual-phase (arterial + portal phase) liver CT to rule out other contraindications for RE, localize the liver tumors, and assess the hepatic arterial anatomy; laboratory investigations and a physical examination to assess vital functions and general condition of the patient.

#### Treatment planning

Eligible patients will be asked to provide informed consent to participate in the study. Next, the interventional radiologist and nuclear medicine physician will make an individualized treatment plan (including identification of the target vessels for selective infusion, identification of extrahepatic branches, and determining the need for coil embolization), based on the anatomy of the hepatic arterial vasculature visualized on pretreatment CT images. In all procedures, two selective injection positions (in the left and right hepatic artery) will be used to treat the whole liver on the same day. In patients with a variant hepatic arterial configuration, intrahepatic arterial branches (for example, the segment 4 artery) may be coil embolized to induce redistribution of blood flow through intrahepatic collaterals, and enable the use of two selective injection positions.

The total amount of ^166^Ho activity (A_Ho166_) required to deliver a whole liver absorbed dose of 60 Gy – the maximum tolerable radiation dose previously determined in a phase I dose-escalation study – is calculated using the formula:

$$ {A}_{Ho166}(MBq)=\frac{60\;(Gy)}{15.87\;x\;{10}^{-3}\;\left(J/ MBq\right)}\;x\; Liver\; weight\;(Kg) $$

Liver weight (in Kg) is determined by multiplying the volume of the liver (measurement based on CT) with the density of liver tissue (1.06 g/cm^3^). The scout procedure always consist of 250 MBq in 60 mg of microspheres (density 1.4 g/cm^3^), this activity is subtracted from the activity given in the treatment procedure. The treatment activity for each perfusion territory is fractionated based on its liver volume. If coil embolization of an intrahepatic branch is planned, the volume of its perfusion territory is included in the side that is anticipated to take over the blood supply.

#### Scout and therapy procedures

All patients will be admitted to hospital (University Medical Center Utrecht, The Netherlands) and will undergo a ^166^Ho scout procedure the next morning, followed by the ^166^Ho therapeutic procedure in the afternoon when the scout procedure was performed successfully.

During the ^166^Ho scout procedure, digital subtraction angiography (DSA) and C-arm CT imaging will be performed to confirm adequacy of the intended injection position(s), and rule out potential extrahepatic shunting. Coil embolization of side branches will only be performed when inevitable to warrant safe treatment. Nitroglycerine 200 μg is given per intra-arterial catheter to prevent vasospasm. Ultimately, a scout dose (250 MBq) of ^166^Ho microspheres will be administered through the anti-reflux catheter (ARC) and the SMC in the target vessels.

Next, a SPECT scan combined with a portal venous phase liver CT will be acquired and evaluated to rule out extrahepatic deposition of ^166^Ho microspheres, assess the intrahepatic biodistribution, as well as the liver-to-lung shunt. In the absence of extrahepatic deposition and a significant liver-to-lung shunt (visible accumulation ^166^Ho in the lung parenchyma), patients will undergo the therapeutic procedure in the afternoon. In the case of an unsuccessful scout procedure, the patient will be scheduled for a repeat procedure if possible.

During the therapeutic procedure, the calculated treatment activity of ^166^Ho microspheres will be administered in the same position(s) as during the scout procedure. Patients are discharged the day after treatment. The posttreatment SPECT (combined with a dual-phase liver CT) is obtained approximately 5 days after treatment to reduce the influence of detector dead time.

#### Follow-up investigations

A telephonic consultation will be scheduled at 2 weeks after treatment to evaluate the patient’s general condition, and ask for the occurrence of adverse events. One month after treatment, patients will undergo a physical examination and laboratory investigations for toxicity assessment. At 3-month follow-up, this is complemented by a whole-body ^18^F-FDG-PET + dual-phase liver CT for tumor response assessment, after which the study follow-up is completed.

### Study objectives

The primary objective is to assess the difference in posttreatment T/N activity ratio on SPECT/CT between administration with the ARC and SMC.

Secondary objectives include comparison of the following endpoints between administrations with the ARC and SMC: mean tumor and healthy liver absorbed dose on SPECT/CT, percentage of calculated treatment activity administered (infusion efficiency), predictive value of the ^166^Ho scout dose distribution, and posttreatment tumor response. Furthermore, the presence of a dose-response relation between tumor absorbed dose and posttreatment tumor response will be evaluated.

### Outcome assessment

In every target vessels’ perfusion territory, the absorbed dose on ^166^Ho-SPECT reconstructions will be determined for the metastases and the healthy liver tissue [4]. Consequently, a posttreatment T/N activity ratio will be calculated for each perfusion territory by dividing the number of counts in tumorous and healthy liver tissue.

The predictive value of the scout dose will be assessed by comparison of the distribution of the scout dose with the treatment dose. The analysis will be based on tumor and healthy liver absorbed doses on SPECT/CT analysis.

Infusion efficiency is defined as the percentage of the prepared treatment activity that is infused. The ^166^Ho activity that is not infused will be determined by measuring the administration system (vial, lines, and catheters) with a dose calibrator.

Response analysis will be performed in accordance with the Response Evaluation Criteria in Solid Tumors (RECIST) version 1.1 [12], for each functional liver lobe separately. Disease control rates (percentage of liver lobes with complete response, partial response or stable disease) will be determined by CT evaluation at 3 months posttreatment. Metabolic tumor response will be determined by assessing the change (relative to baseline) in total lesion glycolysis values on ^18^F-FDG-PET imaging. During the response assessment, observers will be blinded for the catheter type used.

The relationship between tumor absorbed dose on SPECT/CT and tumor response on both CT and ^18^F-FDG-PET will be characterized on the level of perfusion territories.

Common Terminology Criteria for Adverse Events v 4.03 will be used to describe laboratory and clinical toxicity for the entire cohort, with specific attention for device-related adverse events per catheter type such as the occurrence of reflux, vasospasm, or arterial dissections.

The municipal administration will be consulted to assess the overall survival, measured from treatment onward.

### Statistical considerations

#### Sample size calculation

A subgroup analysis of the T/N activity ratios in seven patients with CRLM, previously treated with ^166^Ho RE using a SMC [3], showed a mean T/N ratio of 1.6 (standard deviation 0.57). Sample size calculation, based on a two-sided paired *t* test for the comparison of T/N activity concentration ratios at an alpha level of 0.05 and power of 0.90, showed that at least 23 patients need to be included to detect a difference of 0.4 in mean T/N ratios (with an estimated standard deviation 0.57) in the ARC arm (estimated mean T/N ratio 2.0) and SMC arm (estimated mean T/N ratio 1.6). This corresponds to a 25 % increase (considered clinically relevant) in favor of the ARC arm. A rounded number of 25 patients will therefore be treated in this trial. Sample size calculations were performed with the computer program ‘PS: Power and Sample Size Calculation’ version 3.0 for MacOsX.

#### Randomization

A computer-generated stratified permuted block randomization with varying block sizes will be used. Difference in tumor burden (<10 % or ≥ 10 %) between the two target volumes in the liver will be used as stratification factors to eliminate the potential influence of tumor burden on the comparison of T/N activity ratios between catheter types.

#### Statistical analysis

A comparison of continuous outcome measures between ARC and SMC infusions, such as the mean T/N activity concentration and mean decrease in total lesion glycolysis at 3 months posttreatment, will be performed by means of a paired *t* test. Categorical outcome measures, such as infusion efficiency and disease control rates at 3 months posttreatment, will be compared with a McNemar’s test. The value of the ^166^Ho scout dose distribution will be assessed per catheter type. Correlation and agreement with the ^166^Ho therapy dose distribution will be evaluated with a linear regression analysis (R^2^) and Bland-Altman analysis (limits of agreement), respectively. The dose-response relationship will be evaluated by linear regression analysis. Survival analysis by the Kaplan-Meier method will be used to estimate the median overall survival time for the entire cohort. The primary analyses will be performed on an intention-to-treat (as randomized) basis.

A two-sided *p* value < 0.05 will be considered statistically significant.

#### Study organization

Data completeness and accuracy will be frequently checked by the Department of Radiology and Nuclear Medicine, University Medical Center Utrecht. An independent clinical research associate (Julius Clinical, Zeist, The Netherlands) will audit the trial conduct during 3–4 visits. Appointing a data safety monitoring board was not required for this trial. As per Dutch regulations, serious adverse events, serious adverse device effects, and protocol violations are recorded and reported to the institutional review board. There are no stop rules in this study. No interim analysis is planned.

## Discussion

In patients with advanced colorectal cancer, liver metastases are the primary cause of morbidity and mortality. Unfortunately, only a minority of patients is a candidate for surgical resection with curative intent. Patients with irresectable disease will first receive palliative systemic therapy. Though, despite major advances in systemic treatment, overall survival remains disappointing in this subgroup of patients [13, 14].

Current standard of care in the systemic treatment of metastatic colorectal cancer is based on cytotoxic fluoropyrimidines, oxaliplatin, and irinotecan, as well as targeted therapy with the vascular endothelial growth factor (VEGF)-targeted monoclonal antibody bevacizumab [15–19]. There is no clear preference for sequential exposure to these drugs during consecutive lines of treatment or upfront combination therapy [20, 21]. When disease progression or intolerable toxicity occurs during first-line treatment, patients will subsequently receive another regimen as second-line treatment, with the choice of the regimen depending on the first chemotherapeutic agents. In the Netherlands, the first-line regimen of choice is CAPOX-B (capecitabine, oxaliplatin, and bevacizumab). After initial treatment with six cycles of CAPOX-B, maintenance therapy with capecitabine and bevacizumab is given until disease progression [22]. Subsequently an irinotecan-based regimen, e.g., FOLFIRI (leucovorin, fluorouracil (5-FU), and irinotecan) or irinotecan monotherapy, is indicated as second-line therapy. Only patients with a KRAS wild-type tumor may benefit from additional (third-line) treatment with an epidermal growth factor receptor (EGFR)-targeted monoclonal antibody (panitumumab or cetuximab).

Liver-directed therapy such as RE is increasingly applied as an alternative to achieve local disease control. Currently, two types of yttrium-90 microspheres are used in worldwide clinical practice: resin (SIR-Spheres, SIRTeX, Lane Cove, Australia) and glass (TheraSphere; BTG, Ottawa, ON, Canada) yttrium-90 (^90^Y) microspheres. In salvage patients with colorectal liver metastases, who have no regular treatment options left and an average life expectancy of less than 6 months, median overall survival after RE treatment with ^90^Y microspheres is around 12 months when given as monotherapy or in combination with chemotherapy [1]. Besides, treatment is generally well tolerated, with typical clinical toxicity being limited to mild symptoms of fatigue, abdominal pain, nausea, vomiting and/or fever during the first 2 weeks after treatment [23].

Despite these benefits of RE, there is still room for improvement. Unintentional deposition of radioactive microspheres in tissues other than the liver may cause serious treatment complications. Therefore, a safety procedure is performed in the week(s) before the actual treatment. During this procedure, coil embolization of extrahepatic branches may be performed and a strategic catheter position is chosen before administering a (harmless) scout dose of technetium-99 m-labelled macro-aggregated albumin (^99m^Tc-MAA). Afterward, SPECT/CT and planar nuclear scintigraphy are obtained to exclude the presence of extrahepatic activity and significant liver-to-lung shunting. The treatment procedure is typically performed 1–2 weeks later, with the administration of ^90^Y microspheres from identical catheter positions, followed by posttreatment imaging with bremsstrahlung SPECT/CT or ^90^Y-PET/CT [24].

As a second topic of possible improvement, the intrahepatic distribution of therapeutic ^90^Y microspheres cannot be accurately predicted in advance. The scout dose of ^99m^Tc-MAA particles differs markedly in embolic effect, size, weight, and number of particles infused [25], and therefore fails to predict the intrahepatic distribution of ^90^Y microspheres in most cases [26–28]. Besides, imaging of the ^90^Y microspheres biodistribution itself is already a challenge due to the lack of γ-radiation emission. Traditionally, bremsstrahlung SPECT/CT has been used for posttreatment imaging, but it suffers from a low spatial resolution. Internal-pair production-based ^90^Y-PET/CT has become available as an alternative for quantitative imaging, but the low count rate and inherent noise limit its applicability in daily clinical care [29–31].

Third, it is generally assumed that the preferential arterial vascularization of liver tumors will lead to a selective targeting of tumorous tissue following intra-arterial infusion of radioactive microspheres. It is known from pathological examinations of livers treated with ^90^Y RE that radioactive microspheres cluster preferentially within the peripheral tumor vasculature. The concentration of microspheres can be up to 200 times greater in the tumor periphery than in the tumor center and the healthy liver tissue [32]. Various studies have investigated dose-response relationships in RE. The majority of these studies found strong associations between T/N ratios, absorbed radiation doses, tumor response and overall survival [27, 33–39]. Yet, the degree of tumor targeting, as expressed by the T/N microsphere uptake ratio, shows marked interindividual variability in practice, with a reported range of 0.6–25.9 [40]. This heterogeneity in T/N uptake ratios is likely a result of various factors, including differences in tumor angiogenesis, microsphere characteristics, catheter position, and flow-bound distribution physics [25, 33]. A recent investigation demonstrated that up to 60 % of patients with liver metastases treated with ^166^Ho RE had at least one tumor that received less than or an equal amount of radioactivity as compared to the surrounding healthy liver tissue (T/N ≤ 1) [4]. Flamen et al., also reported similar findings, with 38 % of the metastatic liver lesions in their study having an unfavorable T/N uptake ratio (<1) after RE with ^90^Y microspheres [39]. Since unfavorable T/N uptake ratios cannot be predicted and only become apparent after treatment, timely adjustments in treatment technique are not yet feasible.

The highly variable tumor targeting is an important clinical problem that may at least explain some of the inconsistencies in reported tumor response rates after RE [1, 41]. Considering the reported dose-response relationship, it can be expected that improved T/N ratios will positively affect tumor response after RE. It may also reduce hepatotoxicity, since healthy liver tissue absorbed dose has previously been correlated to biochemical toxicity [37]. Improvement of T/N ratios is especially important in CRLM, since metastases from this tumor type are relatively hypovascular compared with other tumor types (such as neuroendocrine tumors or uvea melanoma), and generally exhibit low T/N ratios.

The above outlined shortcomings of current RE practice are being addressed in the SIM trial, for which trial accrual has started as of November 2014. The distinctive imaging capacities and availability of an identical scout dose of ^166^Ho microspheres, combined with the promising effects on particle fluid dynamics facilitated by the ARC, may result in an optimized treatment technique of RE in patients with CRLM.

### Trial status

Patient recruitment was ongoing at the time of submission.

## Additional file

Additional file 1: Standard Protocol Items: Recommendations for Interventional Trials (SPIRIT) 2013 checklist: recommended items to address in a clinical trial protocol and related documents. (DOC 121 kb)
